# Breathlessness

**DOI:** 10.1111/resp.14426

**Published:** 2022-11-30

**Authors:** Peter G. Gibson

**Affiliations:** ^1^ School of Medicine and Public Health The University of Newcastle New Lambton New South Wales Australia; ^2^ Department of Respiratory & Sleep Medicine Hunter Medical Research Institute, John Hunter Hospital New Lambton New South Wales Australia

**Keywords:** breathing, breathlessness, dyspnoea

Breathing is making a comeback. Not that it went away, just that we lost interest. And I cannot see that we are leading that comeback, if anything, we might be getting sidelined, left out in the cold for what is our raison d'etre. Get this. A science journalist writes a book about Breath. Two hundred eighty pages in smallish font. And it's a hit. When it is released in May 2020, *Breath* (https://www.penguin.com.au/books/breath-9780241289129) spends over 18 weeks on the *New York Times* best seller list. What does he have to say about us? Not much, and what he says is not flattering: the way we breathe, our breathing technique, ‘is disregarded by the medical discipline that deals with the lungs and the respiratory tract.’ Ouch!

**Figure:**
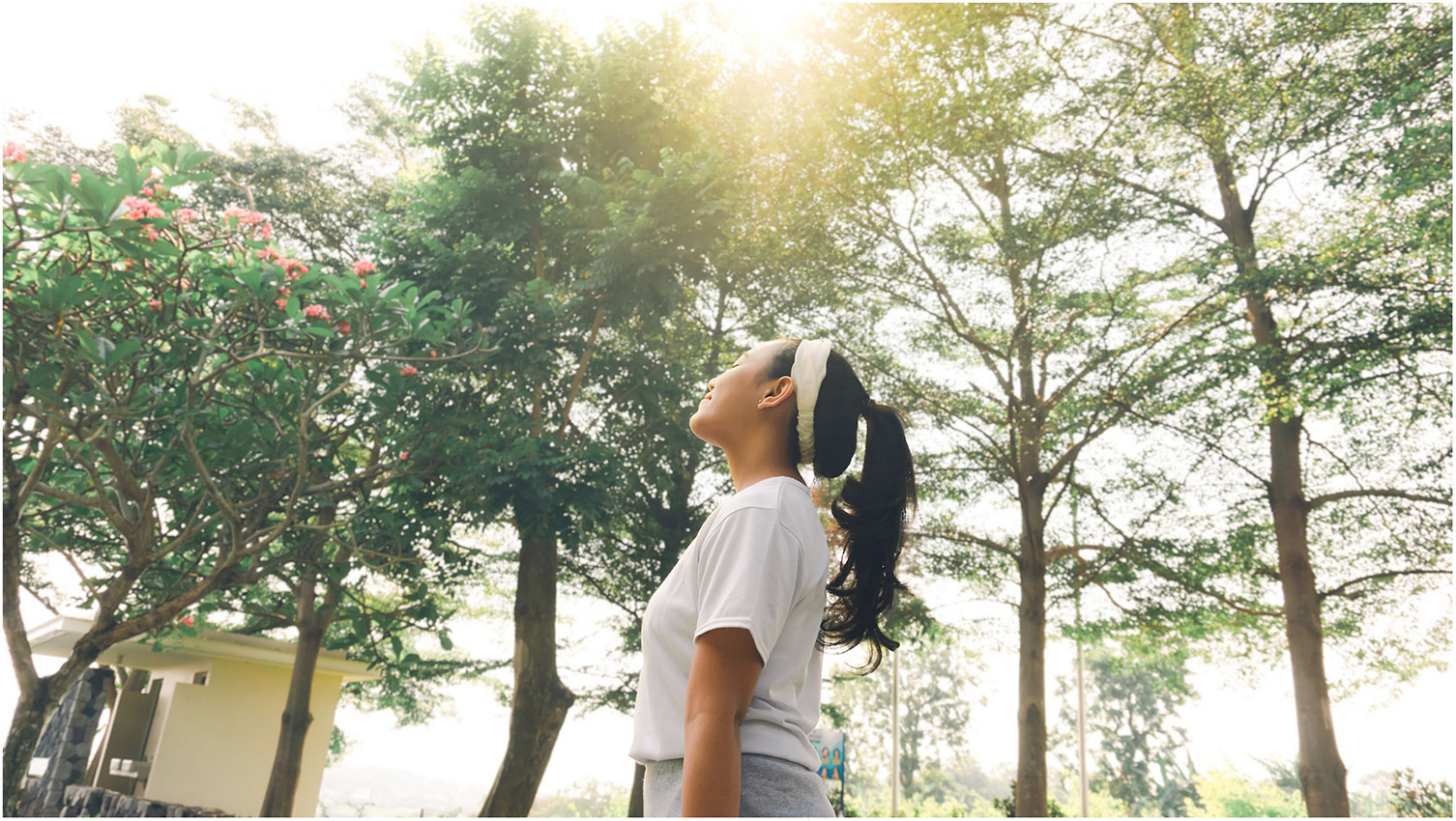
Photo by Gading Ihsan on Unsplash.com

Is that fair? Primary and secondary care physicians respond to scenarios of severe and disabling pain or dyspnoea in patients with severe COPD (https://erj.ersjournals.com/content/52/3/1800887). They are more likely to address chronic pain than breathlessness. Ninety percent do not see a need for further intervention for breathlessness, and less than one in 20 suggest specific breathlessness treatments. Our patients are telling us of their distress but we may not be listening. It seems like we are dismissing breathlessness too easily.

How would you go in this situation? We are good at prescribing inhalers, and assessing and teaching optimal inhaler technique, recommending a slow and steady breathing pattern or a quick and deep one based on the type of inhaler device. But can you assess optimal breathing pattern? And what are your skills in teaching a breathing technique that might relieve breathlessness? There are many situations when we could use those skills: severe COPD, asthma, the terminal phases of any chronic respiratory disease, or the person with unexplained breathlessness.

The need for good *dyspnoea care* is increasing: breathlessness is presenting as a dominant symptom with obesity, and now in long COVID. The National Breathlessness Survey found that obesity accounts for a quarter of breathlessness symptoms in Australian adults (https://doi.org/10.1111/resp.14400). In Long COVID, breathlessness is a common symptom, often occurring with preserved lung function but reduced exercise capacity (https://doi.org/10.1186/s12931-021-01814-9) and displaying features of a disordered breathing pattern (https://bmjopenrespres.bmj.com/content/9/1/e001126).

Many of the effective breathing techniques are accessible and able to be used in a clinician's office or in a rehabilitation setting: for example, breathing training, pacing and prioritizing activity, a hand‐held fan, relaxation, and other cognitive techniques.

Can we go beyond this, and use breathing as part of our own health maintenance, like we do for diet and exercise? It's not as crazy as it sounds. Athletes ‘hack’ their breathing pattern for optimal performance, whether it's the big wave surfer or free‐diver who trains breath‐holding, or the professional marksman who learns to pull the trigger at the end of exhalation. Many of the chronic symptoms attributable to ‘our busy lifestyle’ could be managed by breathing interventions.

So, breathing really is making a comeback. It might be time to upskill and do a bit of breathing ourselves!

## CONFLICT OF INTEREST

None declared.

